# Suicide Prevention Interventions and Their Linkages in Multilayered Approaches for Older Adults: A Review and Comparison

**DOI:** 10.3389/fpubh.2022.842193

**Published:** 2022-05-10

**Authors:** Tomoe Sakashita, Hirofumi Oyama

**Affiliations:** Department of Social Welfare, Faculty of Health Sciences, Aomori University of Health and Welfare, Aomori, Japan

**Keywords:** suicide rate, incidence, universal, selective, indicated, linkage, multilayered

## Abstract

Multilayered approaches to suicide prevention combine universal, selective, and indicated prevention interventions. These approaches may be more successful in reducing suicide rates among older adults if they link these layers more systematically: that is, if the programs are designed so that interventions at a lower level facilitate involvement at a higher level when appropriate. This study aimed to examine the effect on suicide rates of the structure of multilayered approaches, and in particular the types of interventions and the connections or linkages between them. We also wished to consider any different effects by sex. A literature search used PubMed and PsycINFO to identify systematic reviews of interventions in this age group. From the reference lists of these articles, we identified controlled studies assessing the impact of a multilayered program on suicide incidence among older adults. We were particularly interested in initiatives linking different kinds of prevention interventions. We found three relevant systematic reviews, and from these, we identified nine eligible studies. These included seven non-randomized controlled studies from rural areas in Japan (average eligible population: 3,087, 59% women, average duration: 8 years). We also found two cohort studies. The first was from a semi-urban area in Padua, Italy (18,600 service users, 84% women, duration: 11 years). The second was from urban Hong Kong, with 351 participants (57% women) over a 2-year follow-up period. We used a narrative synthesis of these studies to identify five different multilayered programs with different forms of connections or linkages between layers. Two studies/programs (Italy and Hong Kong) involved selective and indicated prevention interventions. One study/program (Yuri, Japan) included universal and selective prevention interventions, and the final six studies (two programs in northern Japan) involved linkages between all three layers. We also found that these linkages could be either formal or informal. Formal linkages were professional referrals between levels. Informal linkages included advice from professionals and self-referrals. Several of the studies noted that during the program, the service users developed relationships with services or providers, which may have facilitated movements between levels. All five programs were associated with reduced suicide incidence among women in the target groups or communities. Two programs were also associated with a reduction among men. The study authors speculated that women were more likely to accept services than men, and that the care provided in some studies did less to address issues that are more likely to affect men, such as suicidal impulsivity. We therefore suggest that it is important to build relationships between levels, especially between selective and indicated prevention interventions, but that these can be both formal and informal. Additionally, to reach older men, it may be important to create systematic methods to involve mental health professionals in the indicated prevention intervention. Universal interventions, especially in conjunction with systematically linked indicated and selective interventions, can help to disseminate the benefits across the community.

## Introduction

Suicide is an important public health issue around the world, particularly among older people (those aged over 60 years) (1). Both suicide rates (2, 3) and the lethality of suicidal behavior (4, 5) are higher in this age group.

A framework has previously been created to identify effective interventions, align them with risk level for suicide and size in the target population, and classify them into three kinds of prevention interventions, universal, selective, and indicated (6, 7). Universal prevention strategies are applied to an entire population or subgroups that have not been identified based on individual risk of suicide. Selective prevention strategies are aimed at groups at risk of suicide, but not necessarily showing suicidal behavior. Indicated prevention strategies focus on high-risk individuals, such as those displaying early signs of suicide potential or who have previously attempted suicide (2).

Suicide risk at individual level fluctuates over time (8, 9), so efforts to reduce mortality from suicide among older adults living in the community need to work with those at various levels of risk. The use of combinations of two or three levels of prevention interventions, known as a “multilayered” approach (6, 10, 11), can both address the level of risk and consider target populations (12). Research suggests that suicide incidence in older adults could be reduced through the use of multilayered approaches (10, 13). Recent community-based multilevel programs involving intervention components of the three kinds of prevention strategies (14–16), for example, used this approach, although there was no reported outcome of a relationship between these components. However, the results of applying this approach to suicide incidence in adults of all ages showed subtle (15, 17) or no visible findings (18). They have also been inconsistent among sites in a study (19) and with a replication study (20). The discrepancy in these results cannot be explained solely by age differences in the target groups or differences in the acceptability and feasibility of large-scale implementation. However, they might be influenced by the structure of the program, and particularly whether it used a multilayered approach to choose and link intervention components. The original framework simply defined the three kinds of prevention interventions by risk level and size in the target population (6, 7). It did not mention linkages between layers. Research about multilayered approaches for suicide prevention has therefore focused on combinations of types of prevention interventions, but paid very little attention to the linkages between these interventions.

**Figure 1:**
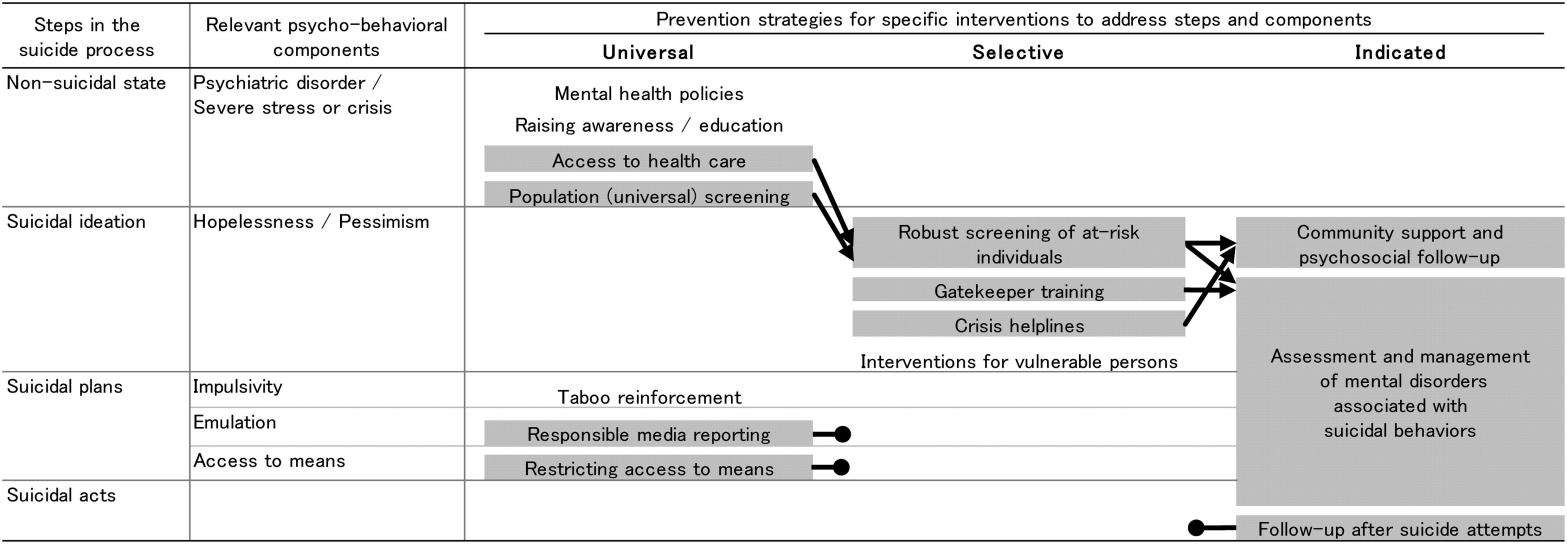
Schematic diagram of suicide process and prevention strategies (22). Interventions highlighted in gray are supported by evidence of their efficacy in reducing suicide risk. A black arrow indicates a clear link to another intervention. A black circle indicates no known link to other interventions. The interventions at each point in the suicide process are expected to involve people at stages closer to suicide.

A previous review has proposed a schematic diagram showing the relationship between the suicide process and prevention strategies, combined with initiatives for linking from universal to selective and from selective to indicated prevention interventions (21). Another review has suggested that some interventions may be successful in reducing suicide rates in older adults because they link universal, selective, and indicated interventions in a structured way (Figure 1), even where each intervention in isolation may not be successful (22). However, it is also clear that some single interventions can be successful without these linkages (2, 23–25). Linking interventions and levels may mean that preventive action can follow individual trajectories toward suicide more closely than separate interventions, and therefore alter these trajectories more effectively.

A multilayered approach to suicide prevention means that there are links between different kinds of prevention interventions, and that these links facilitate movement between interventions. Those who were exposed to a universal or selective prevention intervention, and who are considered to be at higher risk of suicide, can therefore subsequently be involved more easily in a selective or indicated prevention intervention. It is important to ensure that any participants in universal or selective prevention interventions can be seamlessly supported toward a higher level of prevention intervention if their risk of suicide increases. We suggest that making better, more systematic linkages between different kinds of prevention interventions may help with this, by creating a structured way to connect prevention interventions to reduce dropout. However, the coexistence of different kinds of prevention interventions and linkages in multilayered approaches may have different effects from the existence of these interventions without linkages (21, 22). The effects may also vary in different population groups, or by age, sex, and type of suicide outcomes (suicidal ideation, attempted suicide, and death by suicide) (13, 26, 27).

This paper therefore describes a review of studies assessing the impact of multilayered suicide prevention programs on death by suicide among older adults (those aged 60 years and over). It aimed to answer the research questions:

- How are universal, selective, and indicated interventions linked with each other in the multilayered programs reviewed?
- Is there a relationship between program structure, and particularly the type of interventions and the linkages between them, and impact on death by suicide among older adults?
- Does sex have any moderating effects on this relationship?

This review therefore builds on previous work by increasing the focus on the effect of intervention type, and more importantly, linkages between interventions, on suicide in older adults. It sets this within the conceptual framework of earlier work setting out a model linking interventions across levels of intervention with steps in the suicide process (22). This review adds information about the type and role of linkages between interventions. It also aimed to contribute evidence on sex differences in treatment responses. However, it also recognizes that gender expectations and experiences play a significant role in suicide rates, and that these experiences may vary with culture and location.

## Methods

**Figure 2:**
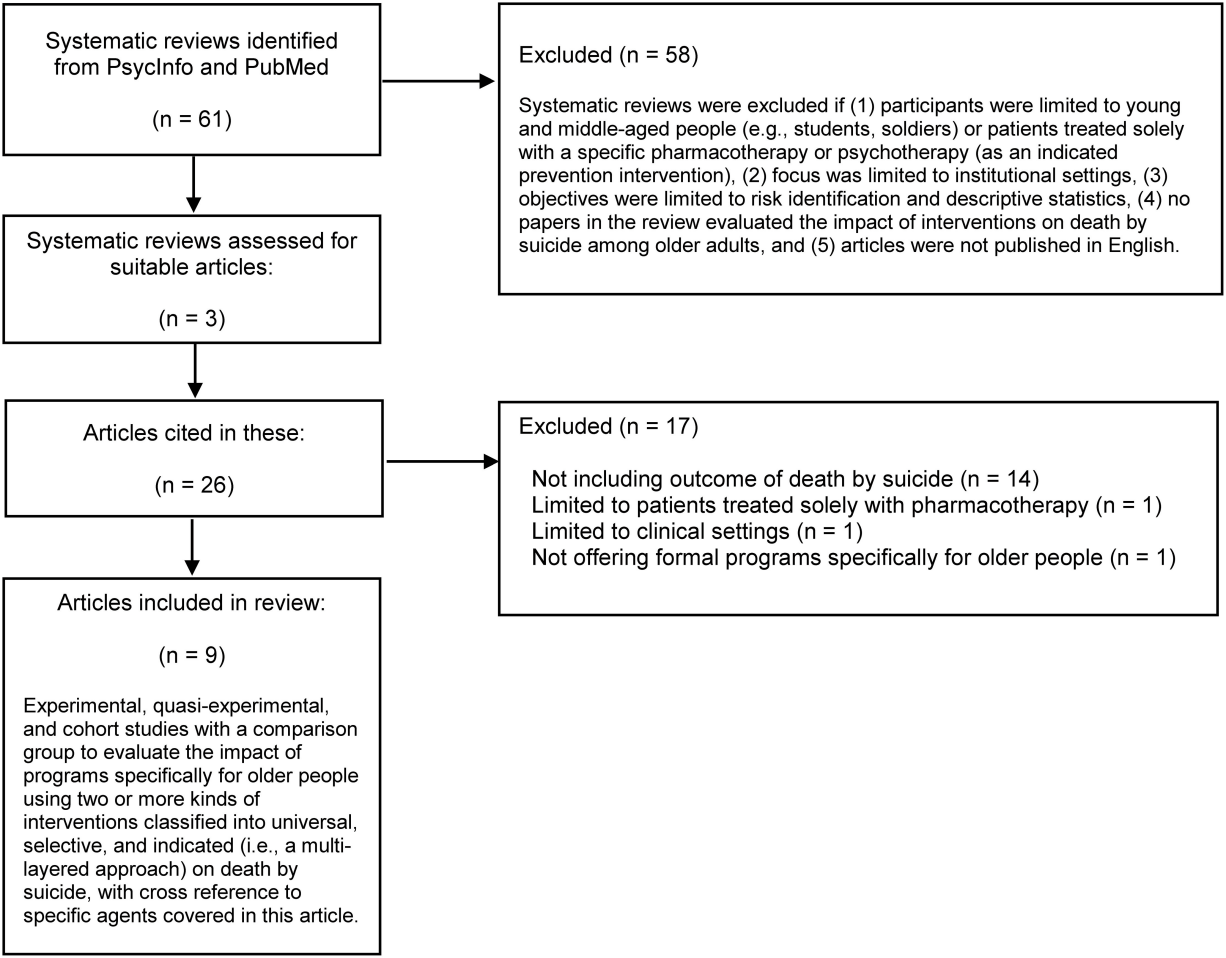
Flow diagram of article selection process.

We wanted to know whether using multilayered approaches to suicide prevention, with an organized structure characterized by specific intervention components and linkages, was related to impact on suicide incidence. We used a literature search to identify systematic reviews as reliable sources of articles on intervention types and linkages (see Appendix 1). We included studies assessing the impact of suicide prevention interventions on suicide incidence in older adults, particularly initiatives linking different kinds of prevention interventions. Figure 2 shows the process of article selection for the review.

We used narrative synthesis because this approach to literature reviews explicitly allows the presentation of statistical outcome data alongside a textual description. This provides opportunities to answer a wider range of research questions. In this case, it allowed us to explore the structure of the intervention program as well as the outcomes and effects of the intervention.

We examined programs evaluated in previous studies to identify all the potential components that served as stand-alone interventions and involved targeted individuals. We used the framework (6, 7) to categorize the intervention components into universal, selective, and indicated prevention strategies. We then reread the studies to identify whether there were linkages between interventions at different levels of prevention and if so, how the intervention components were connected. This enabled us to expand the perspective to program structures characterized by both intervention components and linkages, and examine effectiveness of interventions in light of the program structure.

The review focused on the intervention components identified, the type of prevention strategy, and linkages between levels. We also extracted information from all publications on design, target population, levels of intervention and outcomes (see Table 1). The final step of the review was to consider (a) the components in specific prevention strategies and their linkages within the program structure, (b) any evidence about the effectiveness of the program, individual components and linkages on suicide incidence, if possible, stratified by sex, and (c) any relationship between the program structure and the outcomes.

**Table 1:** Studies included in the review.

**Authors**	**Study**	**Design**	**Target population**	**Level of interventions**	**Outcome**
DeLeo et al. (28)	Tele-Help/Tele-Check in Italy	Large cohort study comparing with the general population	Older users of service (higher proportion of women), living in the community	Selective and indicated	Lower suicide rate than expected among users. No difference between observed and expected suicide rate in men.
Chan et al. (29)	Elderly Suicide Prevention Program (ESPP) in Hong Kong	Cohort study comparing with a historical control	Older adults with a previous suicide attempt, living in the community	Selective and indicated	Greater reduction in suicide rates among suicide attempters compared with control and region. No difference in the reduction between men and women.
Oyama et al. (30)	Group activities in Yuri, Japan	Quasi-experimental study, comparing with a neighboring area	Older adults living in the community	Universal and selected	Greater reduction in suicide rates among women in target area compared with control and region. No change in suicide rates in men.
Oyama et al. (31); Oyama et al. (32); Takahashi et al. (33)	Community-based depression screening with follow-up by psychiatric professionals in three municipalities in northern Japan	Three quasi-experimental studies using the same design in three different areas, comparing with a neighboring area	Older adults living in the community	Universal, selective and indicated	Greater reduction in suicide rates among women and men in target areas compared with control in meta-analyses (21).
Oyama et al. (34); Oyama et al. (35); Oyama et al. (36)	Community-based depression screening with follow-up by primary care professionals in three municipalities in northern Japan	Three quasi-experimental studies using the same design in three different areas, comparing with a neighboring area	Older adults living in the community	Universal, selective and indicated	Greater reduction in suicide rates among women in target areas compared with control in a meta-analysis (21). No difference in change in suicide rates among men between target areas and control in a meta-analysis (21).

## Results

We identified 61 review articles and excluded 58 because they did not meet the criteria (Appendix 1). This left three systematic reviews (13, 37, 38) for further examination, and their reference lists gave nine suitable studies to examine the effect of program structures on death by suicide rate (see Figure 2).

These nine studies described five different multilayered approaches meeting our criteria:

1. Tele-Help/Tele-Check in Italy, a system of crisis helplines and telephone counseling by trained staff (28);
2. The Elderly Suicide Prevention Program (ESPP) in Hong Kong, with community support provided by primary care gatekeepers and psychogeriatric care management for suicide attempters (29);
3. Group activities in Yuri, in Japan, based in the community and run by health professionals (30);
4. Three suicide prevention programs in Sanpachi, Joboji and Matsunoyama, in Japan, which all consisted of community-based depression screening (CDS), followed by subsequent support from mental health professionals (31–33); and
5. Three suicide prevention programs in Yasuzuka, Matsudai and Nagawa in Japan, involving community-based depression screening (CDS) and subsequent support from primary care professionals (34–36).

All nine studies provided an intervention program for older adults living in the community and evaluated it in a community setting. Table 1 shows an overview of the multilayered prevention approach in each, plus the source, design, target population, intervention type and linkage, and effect on suicide death among older adults. Table 2 provides more detailed information about the study areas, including the suicide rates nationally and in those areas, and the population that was eligible for the study. Finally, Appendix 2 provides detailed outcomes, including by age group and sex where available.

**Table 2:** Demographic and suicide data for the studies, including nationally and for the study areas.

**Country (region/city) (ref)**	**Group**	**National suicide rates per 100,000, men**	**National suicide rates per 100,000, women**	**Year to which figures relate^**†**^**	**First year of intervention**	**Age and group for study baseline**	**Target population**	**Eligible population (average annual) or study participants**	**% women**	**Baseline suicide rates per 100,000, study area, men**	**Baseline suicide rates per 100,000, study area, women**	**Length of imple-mentation period, years**
Italy (Padua) (28)	65–74 years 75+ years	22.1 44.6	8.1 8.6	1993	1988	≥ 65 years in 1988–1998	Service users ≥ 65 years, living in the community	18,600 service users	84%	58.9	18.6	11
Hong Kong (29)	65–74 years 75+ years	26.1 44.0	12.9 31.9	2002	2002	≥ 65 years during 2001–2002 in a historical control	People ≥ 65 years with a previous suicide attempt, living in the community	351 study participants	57%	0.06*	0.02*	5
Japan (Yuri) (30)	65–74 years 75+ years	30.4 53.2	19.4 34.4	1995	1995	≥ 65 years during baseline period in target area	People ≥ 65 years living in the community	1,601	59%	241	318	8
Japan (Sanpachi) (31)	60–74 years 75+ years	50.1 45.2	19.9 24.6	2003	2005	≥ 60 years during baseline period in target areas	People ≥ 60 years living in the community	14,578	59%	127	59	2
Japan (Joboji) (32)	65–74 years 75+ years	36.6 63.0	25.3 48.5	1990	1990	≥ 65 years during baseline period in target area	People ≥ 65 years living in the community	1,303	59%	273	316	10
Japan (Matsunoyama) (33)	65–74 years 75+ years	42.6 74.8	29.7 54.3	1985	1985	≥ 65 years during baseline period in target area	People ≥ 65 years living in the community	1,163	59%	242	420	7
Japan (Yasuzuka) (34)	65–74 years 75+ years	36.6 63.0	25.3 48.5	1990	1991	≥ 65 years during baseline period in target area	People ≥ 65 years living in the community	1,190	57%	335	249	10
Japan (Matsudai) (35)	65–74 years 75+ years	36.6 63.0	25.3 48.5	1990	1988	≥ 65 years during baseline period in target area	People ≥ 65 years living in the community	1,333	58%	270	326	10
Japan (Nagawa) (36)	65–74 years 75+ years	46.1 60.7	22.0 34.1	1999	1998	≥ 65 years during baseline period in target area	People ≥ 65 years living in the community	439	61%	371	426	6

† *The year for each set of figures was the closest to the first year of implementation period that was available.*

* *Absolute incidence of suicide in the study group, not rate per 100,000*.

One of the nine studies (28) was a large prospective cohort study comparing the target population with the general population, one was a medium-sized cohort study with a historical control (29), and seven were quasi-experimental studies with matched concurrent controls (30–36). The next few sections describe each approach in turn, setting out the content of each program, its impact, and the type of interventions and linkages between them. We then integrated the findings to explore the relationship between program structure, characterized by the intervention components and linkages, and the outcome.

### Tele-Help/Tele-Check Program in Italy

A pioneering Tele-Help/Tele-Check program was established in 1988 to overcome older people's unwillingness to use phone-based services, in the Veneto region of Italy (28). Suicide rates among older people in that region at the time were much higher than the national average (Table 2). The service was delivered for 11 years, and involving service users living in the community. No ethnic information was provided about the study participants. However, the study gave information by sex about the marital status and living situation of the participants (Appendix 2).

#### Content of Program

General practitioners (GPs) or social workers from local health services typically initiated referrals to the Tele-Help/Tele-Check service. Following referral, clients were placed on a waiting list and subsequently contacted following regional government authorization. Service users received an alarm device that could remotely trigger a pre-established response network. This provided a rapid response in medical and psychological emergencies (Tele-Help). Users also received welfare monitoring and emotional support from trained and paid staff, via short and informal twice-weekly telephone interviews (Tele-Check). Users were able to initiate calls at any time.

#### Impact of Program

Over the 11 years during which the service was delivered, there were fewer deaths by suicide than expected among 18,600 users aged ≥ 65 years (mean age 80 years; 84% women) compared with the general population (2 vs. 11.98 in women, and 4 vs. 8.88 in men). However, the difference for men was not statistically significant. This may have been because of the low levels of suicide in men overall, particularly with the relatively low proportion of men among the service users, meaning that the study had low statistical power.

Tele-Check was the most influential type of support for the 20,000 users of this service. Those using the service for at least 6 months showed reductions in requests for home visits by GPs, hospital admissions, and levels of depressive symptoms.

#### Types of Interventions and Linkages in Program

This program included two main components. The first was a regular phone call by trained staff, which provided assessment and emotional support. This was a selective prevention intervention because the target population was older adults who were socially isolated and functionally impaired. The emergency response and assistance was an indicated prevention intervention, because the target population was older adults who had called for help in a crisis.

There was no formal connection or linkage between the two separate interventions in the structure of the program, except that the participants of the two were the same. However, a case history of an old disabled man recovering from a suicide attempt (39) suggests that the emotional support provided through regular phone contact and the pre-established relationship with the people who would be providing emergency management was an important factor in that case. We speculate that this might foster people's feelings of connectedness to support services, and therefore increase their willingness to use crisis services. This study found an effective structural network in the connection, and this formed a *de facto* systematic linkage between the two prevention interventions. In this study, therefore, users themselves were the connection between the selective and indicated prevention strategies, manifest in an emergency alarm from a user in crisis. No exact data are available, but the implication in the paper is that this connection was used by a substantial proportion of the target population.

### Elderly Suicide Prevention Program in Hong Kong

The Elderly Suicide Prevention Program (ESPP) was launched in 2002 to serve all areas in Hong Kong through seven regional services. The program was directly funded and managed by the Hospital Authority, the only government-funded health management organization in Hong Kong (29). No ethnic information was included about the study participants, although information was provided about their marital status, living situation and educational level (Appendix 2).

#### Content of Program

The ESPP is a multifaceted program including treatment of depression, gatekeeper training, aftercare for suicide attempters, and care management. It operated at two levels: primary and psychogeriatric care services. A typical referral pathway and aftercare would be for a gatekeeper in primary care to identify an at-risk individual displaying clinical depression or suicidal ideation/plans/attempts, and make an urgent referral. Each client was then assigned a care manager, a psychogeriatric nurse who arranged an urgent psychogeriatric appointment within 1 week. At the psychogeriatric clinic, designated psychogeriatricians provided the assessment and any follow-up care that was clinically indicated. The care manager provided frequent phone contacts, regular home visits, and *ad hoc* home visits in response to crises. These were used to monitor clients' mental and social situations, promote compliance with treatment, and provide psychoeducation in the first 6 months. The care manager also liaised with other caregivers. ESPP clients were given emergency contact details for their care managers. A multidisciplinary team including psychogeriatricians and care managers at tertiary care level held regular case conferences to review the care plan and clinical progress.

#### Impact of Program

This was a relatively small observational cohort study. The 351 participants were selected from older adults (aged ≥ 65 years) who had made a suicide attempt and completed a 6-month ESPP. If clinically indicated, they had also received aftercare from a psychogeriatrician (29). The dropout rate from outpatient care after the ESPP was 17.7% during the 2-year follow-up period. The 2-year fatal suicide rate was lower in the study participants than in a historical cohort group, but this difference was not statistically significant among women. Re-attempt rates were similar for the participants and the historical control group. At a population level, suicide rate only dropped significantly in women aged ≥ 85 years from baseline.

The ESPP was therefore associated with a non-significant reduction in the rate of fatal suicide attempts in older adults with a past suicide attempt. It may also have contributed to a fall in the population suicide rate in women aged ≥ 85 years. The authors speculated that the gatekeeper activity in the intervention program (ESPP) resulted in better recognition of depressive illness and enhanced access of the service to at-risk older women (aged ≥ 85 years). However, the non-significance of the results could have been related to both the low number of people in the sample and external environmental issues. For example, the epidemic of severe acute respiratory syndrome (SARS) in the area had resulted in fewer people being able to access mental health services in the region in 2003, and also increased pessimism among the general population. This was thought to have contributed to higher suicide rates in 2003 and 2004, which were included in the intervention period.

#### Types of Interventions and Linkages in Program

This program included four main components. The gatekeeper training for primary care workers and the regular contact with a care manager were both selective prevention interventions. The target population was older adults who had made a past suicide attempt but were not necessarily always at a higher risk level than baseline. Treatment by a psychogeriatrician was an indicated prevention intervention because the target population was people who had been assessed as needing this type of follow-up support. The emergency contact by the care manager was also an indicated prevention intervention because the target population was older adults in a crisis.

The urgent referral to the care manager or psychogeriatric nurse was the connection between the gatekeeper activities of the primary care workers (selective prevention intervention), and the assessment and treatment by a psychogeriatrician (indicated prevention intervention). The care managers effectively provided the linkages between levels throughout the program, both at initial referral, and during any crisis, building on their relationships with the service users. In this study, again, users therefore developed familiarity and an emotional connection with the service. This may have made it easier for them to seek additional support when necessary.

There are no exact data available, but this connection seems to have been used in a substantial proportion of the target population. This may be partly because the participants in the ESPP had a relatively low dropout rate. The ability to access higher level services when the need was urgent, coupled with multidisciplinary activities led by the assigned care manager, enhance the idea that this connection was both structural and pre-planned. Building connections with service users as a way to reduce dropouts could also form a systematic linkage between the two kinds of prevention interventions.

### Group Activities for Older People in Yuri, Japan

This was a community-based program designed to build social relationships among older people. Lack of these relationships is the most common risk factor for late-life suicide. The program was launched in 1995 in a rural area of norther Japan with a high suicide rate. It was funded and managed by the local government (30). The only socio-economic information provided in the paper related to the unemployment rate and average yearly income, both of which were lower than the prefectural average (Appendix 2).

#### Content of Program

This program included group activities and low-threshold campaigns. The group activity was designed to help the participants to develop closer relationships with neighbors and create a more fulfilling social life. It provided opportunities for a target population aged ≥ 65 years, particularly those who were identified as having poor social relationships (e.g., living alone and feeling isolated from their family), to participate in social, voluntary, and recreational activities and to exercise together. Sessions were held once every 2–4 months in the community center in each district within the town, run by municipal public health nurses (PHNs). The PHNs specifically identified people with poor social relationships and encouraged them to join the group activities, and especially to enhance their social relationships. There was also an educational campaign for the general public, involving psychoeducational workshops (run once or twice yearly) and local newsletters to provide information about factors that were protective (e.g., social relationships and activities) and associated with higher risk (e.g., depression) of late-life suicide.

#### Impact of Program

This was a quasi-experimental study to assess the impact of the intervention program on suicide incidence over an 8-year implementation period. All participants were geographically defined, population-based dynamic cohorts of older adults aged ≥ 65 years. The study used a pre–post design with a primary outcome of change in the incidence of death by suicide (suicide rates) between the 8-year baseline and 8-year implementation period, including a non-randomized matched control (average annual population aged ≥ 65 years: 1,601 [59% women] in intervention; 1,933 in control) and the regional prefecture. A greater reduction in suicide rates was observed among women in the intervention area than in the control area and wider region. However, there was no change in suicide rates in men (30). The annual total number of participants in the group activity sessions increased from 232 to 3,051 rapidly during the 8-year implementation period. No data are available on the sex of the participants. At least half of the target population seemed to participate in at least some sessions although no exact data were available. At least one-third or more of the target population seemed to participate in the workshops at least once during the same period.

#### Types of Interventions and Linkages in Program

This program included two components identified as stand-alone interventions. The educational campaign run through workshops and other local media was a universal prevention intervention, because the target population was all older people living in a particular area. The workshops appeared to have a relatively high participation rate. The group activity component was both universal and selective, because it was open to any older people living in the area, but the nurses also specifically encouraged attendance of those at increased risk of suicide because of their poor social relationships.

There were two types of group activity, and the PHNs recommended one or other to participants, depending on the quality of their social relationships. There was therefore both a link between the universal and selective aspects of the program, and a connection between the two types of group activities. This connection seems to have been used by a substantial proportion of the target population, because the group activity had a high participation rate. The connection was not necessarily either structural or pre-planned, but the two types of group activities were in the same setting, so it was not hard for people to move between them. This connection is therefore not likely to be a systematic linkage, but again based on service user familiarity with both location and the people running the session. Both the campaign and group activity components had high participation rates, and it seems likely that some participants would have been involved in both. The educational campaign may therefore have contributed to the rapid increase in the number of participants in the group activities, although no direct connection was reported between the two.

### Community Depression Screening and Follow-Up Support Among Older People in Northern Japan

Six of the nine studies included in the review assessed the impact of community-based depression screening (CDS) interventions on suicide incidence among older adults, in a quasi-experimental design. Participants were geographically defined, population-based dynamic cohorts of older adults. The studies used a pre–post design with a primary outcome of change in the incidence of death by suicide (suicide rates) between the baseline and implementation period, and five of the six included non-randomized matched controls.

The six municipal projects autonomously ran a CDS program in northern Japan from the 1980's through to the 2000's in rural areas with high suicide rates among older people. Screening was carried out by PHNs and there were two follow-up methods: support from psychiatrists (CDS with psychiatric care support) in three municipalities (31–33) and support from GPs (CDS with primary care support) in three municipalities (34–36).

Three studies included no demographic or socio-economic information (32, 33, 36). The others provided unemployment rates (31), or both unemployment and average income (34, 35) compared with the regional average (Appendix 2).

#### Content of Program

All six studies included a municipality-based screening process. All target older adults living in the community were invited to participate in a two-step screening program for depression, with the offer of subsequent support. In the first-stage, universal screening, a self-report questionnaire for depression screening was delivered to all target residents aged ≥ 60 or 65 years. The adults who agreed to participate returned the completed questionnaire. In the second stage, adults who were screened as positive for depression from the self-report questionnaire were advised to take part in semi-structured face-to-face and telephone interviews with a public health nurse or psychiatric social worker. This provided an assessment using diagnostic criteria. After these interviews, a tentative diagnosis was made with input from psychiatrists or GPs, and the participants were referred to psychiatric or primary care support services, which then provided support and care. The participants were unable to choose their care support pathway. If a formal assessment led to the diagnosis of a depressive episode, a clinical decision was made to encourage visits and regular telephone contact with a public health nurse or psychiatric social worker for 2 months, or to refer the individual for further treatment with a psychiatrist or a GP. The psychiatrists might prescribe antidepressants, benzodiazepines, and/or brief supportive psychotherapy, depending on what they felt was required. The GPs only prescribed benzodiazepines and psychiatric consultations, and not antidepressants or psychotherapy. All interventions were supplemented by health education for the general public conducted via local newsletters and workshops. These were designed to increase awareness about depression and minimize the associated stigma.

#### Impact of Program

The screening was offered to the entire eligible older population aged ≥ 65 years in the five small studies (average of annual populations aged ≥ 65 years: 439–1,333, 57–61% women) and one-third of those in the large study (the population: 11,920, 59% women). The results suggested good uptake of first-stage screening, with maximum annual participation rates of almost 80% during the 6–10-year implementation periods in the five small studies, and an uptake of 52% during the 2-year implementation period in the largest study. The participation rates in screening were only available by sex in two studies. In Sanpachi (31), they were similar for men and women (53 and 51%). In Nagawa (36), the annual uptake of screening ranged from 80 to 95% in women and 60 to 89% in men. In the second stage of screening, maximum annual participation rates were almost 80% in three of the small studies as well as in the large study and were ~60% in the remaining two small studies. The performance of the two-step depression screening process seemed to vary with both the care pathway followed and the size of the target population. The maximum annual detection rate of depressive episodes via psychiatric care support was around 4% of the total screened population in two of the smaller studies and 0.7% in the large study. When using primary care support for detection, the rate was 1.2–2.9% in the three small studies. No data are available by sex for depression detection rates. In all the studies, the total participation rate for workshops during the implementation period was <5% of the total population of the target area.

All three quasi-experimental studies examining CDS with primary care support (34–36) found a decrease in suicide rates among older women in the target areas compared with the control areas (64, 70 and 74%). However, no statistically significant change in suicide rates was observed in older men. The studies examining CDS with psychiatric care support (31–33) found a decrease in suicide rates in both men and women in the target areas compared with the control [51% (not significant), 76% and 60% among women and 61, 73 and 88% in men]. The areas covered by the two sets of studies are very similar. These results therefore suggested the presence of sex differences in the treatment responses for the two types of support pathways.

#### Types of Interventions and Linkages in Program

Both types of CDS programs included four main components. The universal screening through a self-reported questionnaire and the public education campaigns were universal prevention interventions because the target population was all older adults living in the area. The universal screening component achieved high participation rates, but the rates were much lower for the educational component. The second stage screening using a semi-structured interview by a health professional was a selective prevention intervention, because the target population was older adults who were screened as showing depression. The robust screening component also had high participation rates. Subsequent care was an indicated prevention intervention because the target population was older adults who were experiencing a depressive episode and showing signs that they needed additional support or care.

There was a formal link between the universal and selective interventions in the form of invitations to individuals to attend a more robust screening. This invitation was structured to avoid dropouts and resulted in high participation in the robust screening. This can therefore be considered a systematic linkage between them. Information dissemination through the public education work had no direct or indirect connections to other interventions. The education sessions did not appear to facilitate participation in any kind of selective interventions. The formal link from selective to indicated interventions was the referral to subsequent care for individuals with a depressive episode. This referral was again structured to avoid dropouts and resulted in high participation in subsequent care. It can therefore be considered to be a systematic linkage between levels.

## Discussion

Previous studies have found that programs that combine interventions from two or more prevention levels (universal, selective, and indicated) can reduce suicide among older adults. However, it is also possible to achieve reductions in suicide rates from a single prevention strategy without any linkage with other levels, for example, responsible media reporting, restricting access to means, and follow-up care after attempted suicide (2, 23–25). This review therefore sought to examine the reasons why multilayered programs might be effective by looking at the nature of the linkages between levels.

We found nine suitable studies from previous systematic reviews, covering five different multilayered programs. These programs addressed risk predictors such as depression, social isolation, and crisis. All five multilayered programs included linkages between different kinds of prevention interventions. Two involved selective and indicated prevention interventions (28, 29), one involved universal and selective prevention interventions (30), and six studies (two programs) involved linkages between universal and selective prevention interventions, and between selective and indicated prevention interventions (31–36). All five programs were associated with reduced incidence of suicide among older women in the target groups or communities and two programs, ESPP (29) and CDS with psychiatric care support (31–33) were associated with a reduction among both women and men.

**Figure 3:**
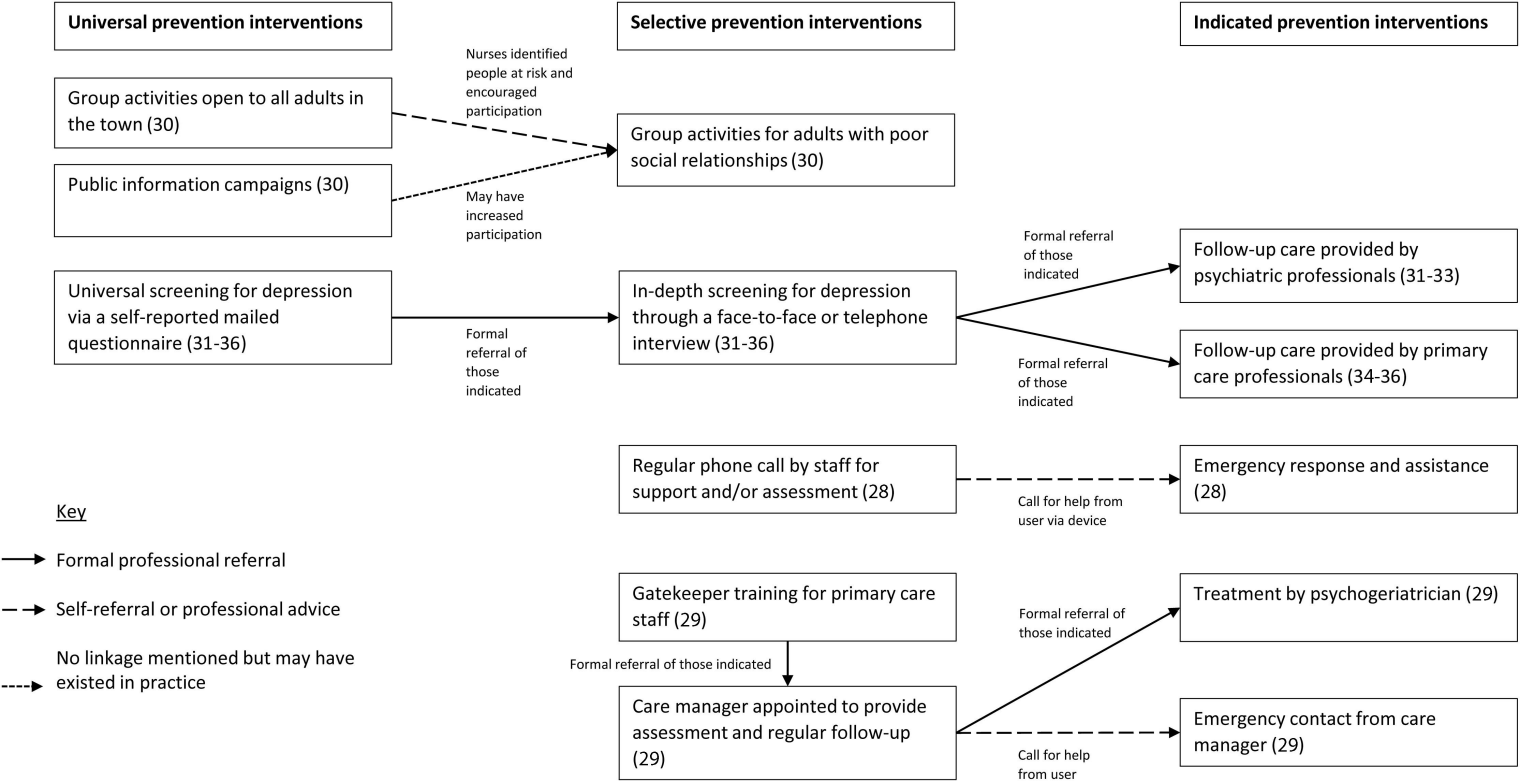
Schematic diagram showing the nature of linkages between elements of the programs included in the review.

Our review suggests that the types of linkages varied between the programs. The linkage in the six CDS studies was a formal referral between levels, which was also possible in ESPP. However, the other programs involved a more informal move between levels, relying on the actions of service users or staff running the programs. This was also possible in ESPP. Figure 3 summarizes these linkages in visual form. The next few sections examine the impact of program structure and the nature of linkages.

### Impact of Program Structure

#### Linkages in the Form of Professional Referrals

Several programs contained formal linkages between levels in the form of professional referrals. For example, ESPP (29) linked selective and indicated interventions through a formal referral by either a primary care practitioner to the formal program (and to a care manager/psychogeriatric nurse) or from the care manager to a psychogeriatrician. This program had an additional element in that it involved people who had previously attempted suicide. Follow-up care among recently discharged patients has been found to be effective in reducing suicide attempts and deaths in all age groups (40), especially at-risk individuals discharged from emergency departments (41). However, this follow-up care has often not been designed to be integrated with other prevention strategies (40, 42), which might limit its benefits. ESPP was therefore interesting in its systematic linkages between selective and indicated prevention interventions for those with a previous history of attempting suicide.

The six studies in northern Japan had formal links across all three levels through referrals. Screening participants whose results suggested depression were invited for a more in-depth assessment. Those assessed as experiencing a depressive episode were referred for follow-up care. In the six studies, these prevention components achieved high participation, and resulted in a lower suicide rate among older adults, confirmed by a recent meta-analysis (21). This is also supported by a longitudinal study that found an association between exposure to screening and lower suicide incidence (43).

The precise reasons why formal referrals between levels are effective is not clear. We speculate that perhaps a referral from a universal screening program overcomes the recognized barrier of having to ask for help. For example, a previous study suggested that mental health screening and feedback improved recognition and encouraged service use (38). However, the effect of universal screening may also be related to the form of contact. Other studies (42, 44) have found that brief contact interventions such as letters, postcards, and telephone calls, could reduce suicide rates. Future research may wish to examine this issue in more depth.

#### Informal Linkages Involving Professional Advice or Self-Referrals

There were two main types of informal linkages seen in the studies in the review. The first involved professional advice, and the second self-referrals.

Some of the studies in our review involved healthcare professionals targeting particular groups or individuals to encourage them to use particular services. For example, in the study involving group activities in Yuri (30), the group activities were open to anyone (a universal intervention). However, when nurses identified people who would benefit from group activities, or even a particular type of group activities, they encouraged them to participate. Social disconnectedness is associated with a greater risk of late-life suicide (45, 46). However, we were able to find only one study that had used increased community participation to minimize social isolation (47), and these results need to be replicated in other intervention studies.

Several of the studies in our review allowed or required service users to self-refer for additional help. Tele-Help/Tele-Check in Italy (28), for example, had a selective intervention of regular telephone calls to service users. However, users were also given a device to use if they had a crisis and needed emergency care. ESPP (29) also enabled service users to refer themselves for additional help in a crisis, as well as having a formal health professional referral route.

#### Impact of Number of Layers in a Multilayered Approach

Conwell and colleagues have pointed out that a multilayered approach that combines three kinds of prevention interventions is probably most effective (48). Our review suggests that there is some truth in this, but that the nature of the linkages also matters. In the study in Yuri (30), there was little linkage between the education component of the study and the more selective interventions. In the CDS studies, the robust screening for depression and subsequent care support by medical professionals probably improved identification and treatment of depression. Gatekeeper training for physicians may have a similar effect (23, 24, 49, 50) in supporting more robust identification of those in need of referral. The strength of the CDS studies was the systematic linkage of selective and indicated prevention components back to a universal element that was seen as useful in the population, resulting in higher participation rates.

### Other Factors That May Affect Impact

The review also identified some other factors that may be significant, beyond the structure of the program. In particular, we identified a common feature of building relationships between service users and healthcare professionals. For example, in ESPP (29) and Tele-Help/Tele-Check (28), clients shared their preferred communication channel for regular and emergency contacts with their support staff. We speculate that using clients' preferred communication channels may help to build relationships and make service users more comfortable with communicating with healthcare professionals. This may help to address the fluctuating nature of suicide risk at individual level and make the linkage more effective over time.

It is also possible that building a relationship with Tele-Help/Tele-Check staff helped to make users more comfortable with asking for additional help in a crisis. However, simply reaching out for help may be insufficient. Another study found that crisis helplines without subsequent support, a selective prevention intervention, can often reduce suicide risk among callers only during the call itself, or for a few weeks at most (51). Appropriate support after a crisis call, as seen with ESPP (29) and Tele-Help/Tele-Check (28), may be necessary to reduce suicide rate in the target group.

### Sex Differences in Treatment Response

Few studies examined sex-specific distinctions in the impact of interventions on suicide incidence. However, those that did suggest that most programs benefited older women more than men (13). Interestingly, those that showed benefits to both men and women were generally those that involved psychiatric or psychogeriatric support, rather than support from nurses or care managers.

For example, ESPP (29) involved identification and treatment of mental illnesses by psychogeriatric professionals. The two groups of three studies examining each type of CDS were also combined in two separate meta-analyses in a study (21). The meta-analyses found an association between both types of CDS and reduction in suicide incidence among older women. However, only CDS with psychiatric care support was also associated with a reduction among older men. A combined analysis across all six studies also suggested the presence of sex differences in the treatment responses for the two types of support pathways (21). These results indicate that involving psychiatric care support after screening or initial identification of those at risk may be beneficial in preventing suicide among men.

Both ESPP and the CDS studies took place in Asian cultures (Hong Kong and Japan), and the findings may therefore not be more widely generalizable. However, a community-based multilevel program to improve early detection and treatment of depression in all age group was conducted in German, using a combination of GP education focusing on male depression, low-threshold campaigns, and psychiatric consultations. This therefore combined selective and indicated prevention interventions with systematic linkages through GPs' management of depression, and a universal prevention intervention in the form of a campaign for the general public (17). This resulted in a lower suicide rate among men of all ages, which we speculate may be related to the combined selective and indicated prevention interventions involving psychiatric consultations.

We suggest that the effect of psychiatric care on men may be because of sex differences in suicide behaviors, and the type of support provided by primary and secondary care. Men appear to be more vulnerable to impulsivity in the suicide process (52, 53). Secondary (i.e., psychiatric and psychogeriatric) care support rather than primary care support provides treatment (e.g., prescribing antidepressants, or hospitalizing people for their own protection) that can ameliorate this impulsivity. Secondary rather than primary care support may therefore be more effective in reducing suicide risk in men (21), although this finding would need to be tested in more cultural settings.

Taken together, these findings suggest that reducing suicide in older men may benefit from including several different elements. First, it may be helpful to involve management of mental illness with suicidal behaviors in the indicated prevention intervention. Second, this may benefit from systematic links from a selective prevention intervention by mental health professionals. This is consistent with the findings of a recent systematic review investigating risk factors for suicide deaths compared with attempts (27). However, future studies will be necessary to confirm these ideas and suggestions.

### Limitations and Suggestions for Future Research

The main weakness of this review was that it used a very small number of studies to examine the issue. Using literature reviews to identify studies was convenient and ensured that only validated studies were examined. However, it may have limited the number of studies available, and particularly excluded very recent studies. Future researchers may wish to use a wider search strategy and include more studies.

There are also very few longitudinal data on the relationship between exposure to particular interventions and program structures, including linkages between interventions, and suicide outcomes, except for one study (31, 43). Future research should address this issue, in a bid to increase the effectiveness of suicide prevention, including through multilayered interventions.

It is also difficult to determine the effective elements when evaluating complex interventions with multiple components. We attempted to address this in this review by considering program structure and the nature of linkages between components. Future studies may wish to examine aspects such as the impact of the number of layers in a multilevel study, and the nature of linkages between levels, including whether they are formal or informal. It would also be interesting to examine the role of relationships and familiarity with services in determining willingness to use services and seek help. Other studies might examine why formal referrals are effective in overcoming barriers to seeking help, and also consider the effect of increased community participation on suicide rates.

Finally, it is difficult to draw firm conclusions about the sex effects of particular approaches. This is partly because of limitations in study design, small sample size, limited number of studies, and heterogeneity of study populations. The studies all included more women than men, especially Tele-Help/Tele-Check in Italy (28), which included over 80% women. This makes it much more difficult to draw conclusions about the effects of the intervention on men, because of the low statistical power of the study for this group.

The likelihood of suicidal behavior will also vary with gender meanings of suicide, and the lifetime experiences of men and women. This means that both gender and sex may affect the likelihood of suicidal behavior. None of the studies in this review distinguished sufficiently between sex and gender to enable these differences to be identified. We suggest that future studies on suicidal behavior should clearly set their findings into the context of gender expectations and meanings related to suicide, and the different experiences of men and women within the cultural settings.

However, our findings also suggest some avenues for future research, especially in preventing suicide in older men. These include examining the importance of involving mental health professionals in indicated prevention interventions for this group, and the effect of linking suicide prevention with managing mental health conditions.

## Conclusion

Studies suggest that community interventions are important in reducing suicide in older adults, and that integrating universal, selective, and indicated prevention interventions may be crucial in this process. Both formal and informal linkages may be effective, including building user familiarity with services. The most important relationship and linkages are probably between selective and indicated prevention interventions. Universal prevention interventions added to combined selective and indicated prevention interventions with systematic linkages may help to disseminate the benefits across the community. The use of sex-specific indicators of treatment response is helpful to identify effective elements of interventions. To increase the effectiveness of suicide prevention programs for men, it may be helpful to include the management of mental illnesses in the indicated prevention interventions. The program structure, characterized by the interventions and linkages, may therefore be a key parameter in determining the effect of multilayered approaches for suicide prevention. Beyond measuring the impact of such strategies on suicide incidence, future studies of multilayered programs are needed to assess their effects on intermediate outcomes related to their structure. To address the potential for prevention, the program structure should be considered in a model that contemplates which intervention can reach individuals at various levels of risk and how each intervention can link formally and informally with other prevention interventions at different risk levels.

## Author Contributions

TS and HO contributed equally to the literature review and manuscript preparation including discussion. Both authors contributed to the article and approved the submitted version.

## Funding

This study was funded by the Ministry of Education, Culture, Sports, Science and Technology of Japan (Grants-in-Aid for Scientific Research No. 17K04208).

## Conflict of Interest

The authors declare that the research was conducted in the absence of any commercial or financial relationships that could be construed as a potential conflict of interest.

## Publisher's Note

All claims expressed in this article are solely those of the authors and do not necessarily represent those of their affiliated organizations, or those of the publisher, the editors and the reviewers. Any product that may be evaluated in this article, or claim that may be made by its manufacturer, is not guaranteed or endorsed by the publisher.
