# Supplementary material for: Suicide Prevention Interventions and Their Linkages in Multilayered Approaches for Older Adults: A Review and Comparison
Source: Front Public Health. 2022 May 10;10:842193. doi: 10.3389/fpubh.2022.842193 (PMC9127334; doi:10.3389/fpubh.2022.842193)
Supplement: Supplementary file 1 [file Data_Sheet_1.docx]

**Appendix 1. Search strategy used to identify suitable studies for inclusion**

We carried out PubMed and PsycINFO searches covering the period from January 2011 to December 2020. The search terms used were suicide AND prevention AND intervention AND (elderly OR older). Function limits were used to identify systematic reviews.

Our inclusion criteria specified systematic reviews evaluating interventions to prevent suicide among older adults living in the community. Studies that clearly did not satisfy the criteria were excluded based on the title and abstract of the article. This included studies where (i) participants were limited to young and middle-aged people (e.g., students, soldiers) or patients treated solely with a specific pharmacotherapy or psychotherapy (as an indicated prevention intervention), (ii) focuses were limited to institutional settings, (iii) objectives were limited to risk identification and descriptive statistics, (iv) no included papers evaluated the impact of interventions on death by suicide among older adults, and (v) articles were not published in English.

These selection criteria allowed us to identify systematic reviews, as a convenient way of identifying all studies on a particular topic. From the reference lists of these articles, we included experimental, quasi-experimental, and cohort studies with a comparison group to evaluate the impact of programs that delivered specifically for older people and adopted two or more kinds of intervention components that can be classified into three kinds of prevention strategies: universal, selective, and indicated (i.e., a multilayered approach) on death by suicide, with cross reference to specific agents covered in this article.

Finally, studies evaluating the impact of a multilayered approach on the incidence of death by suicide (suicide rate) in older adults were chosen from the reference lists of the reviews to verify whether a relation between program structures characterized by the intervention components with the linkages and their impact existed in the multilayered approaches.

**Appendix 2. Further data, definitions and detailed outcomes from the studies included in the review**

| Ref | Country (area) | Type of area | Baseline or control period/ cohort | Time-frame | Definitions used in study (e.g. age cut-offs) | Age groups (%) | Socio-economic info (% of sample) | Outcomes |
| --- | --- | --- | --- | --- | --- | --- | --- | --- |
| 28 | Italy (Padua) | Semi-urban | 1988–1998 | 1988–1998 | Participants were at least 65 years old (mean age 80 years) | No data. | Marital status: 73.10% widowed, 16.80% single, 8.57% married and 1.53% divorced among women; 43.88% widowed, 9.86% single, 43.11% married and 3.15 divorced among men.  Living situation: 74.9% alone and 25.1% with someone among women and 60.3% alone and 39.7% with someone among men. | The observed number of suicides was significantly lower than expected in women (2 vs. 11.98) and non-significantly lower in men (4 vs. 8.88) during the intervention period compared with the general population. |
| 29 | Hong Kong | Urban | 2001–2002 | 2-year follow-up of 2002–2007 | Adults at least 65 years old, who completed a 6-month intervention. | 20% of participants were aged 85 years and over | Marital status: 44.2% married and 54.1% unmarried.  Living situation: 8.8% alone and 91.2% not alone.  Educational level: 85.2% primary or below and 14.2% secondary or above. | Suicide incidence aged ≥ 65 years during 2-year follow-up period in an intervention group: 498 in women and 1,333 in men.  Change in suicide incidence after the intervention: a statistically non-significant 69% lower incidence in women and a significant 79% lower incidence in men in the intervention group compared with control.  Re-attempt rates: 9.97% in the intervention group and 10.6% in the control (no significant difference) during 2-year follow-up. |
| 30 | Japan (Yuri) | Rural | 1987–1994 | 1995–2002 | Adults aged 65 years and over, especially those with poor social relationships | 57.2% aged 65-74 years, 33.2% aged 75-84 years and 9.6% aged 85 years and over during implementation period | Unemployment rate (1.65%) in Yuri was lower than the prefectural mean (3.37%) in Akita in 1995.  Average yearly income (2,630,000 yen) in Yuri was 89.9% of the prefectural mean (2,924,000 yen) in Akita in 1995. | Annual total number of participants in group activity: 232–3,051. No data by sex.  Suicide rates by sex: suicide rates (per 100,000) aged ≥ 65 years during implementation period in target area: 79 in women and 190 in men.  Change in suicide rates (aged ≥ 65 years) following the intervention, from baseline to implementation period: a statistically significant 76% reduction among women and a non-significant 21% reduction among men in target area compared with control. |
| 31 | Japan (Sanpachi) | Rural | 2003–2004 | 2005–2006 | Universal screening for everyone aged 60 years and over. | 37.6% aged 60-69 years, 40.1% aged 70-79 years and 22.4% aged 80 years and over during implementation period | Unemployment rates were 4.98–7.25% in three target municipalities in 2003. | Participation rate in screening, annual %: 51% in women and 53% in men.  Detection rate of depression, annual %: 1.6%, no data by sex.  Participation in workshops: no data.  Suicide rates by sex: suicide rates (per 100,000) aged ≥ 60 years during implementation period in target areas: 29 in women and 50 in men.  Change in suicide rates (aged ≥ 60 years) following the intervention, from baseline to implementation period: a statistically non-significant 51% reduction among women and a significant 61% reduction among men in target areas compared with control. |
| 32 | Japan (Joboji) | Rural | 1980–1989 | 1990–1999 | Universal screening for everyone aged 65 years and over. | 63.0% aged 65-74 years, 29.0% aged 75-84 years and 7.9% aged 85 years and over during implementation period | No data. | Participation rate in screening, range of annual %: 25–78%, no data by sex.  Detection rate of depression, range of annual %: 1.5–3.5%, no data by sex.  Participation in workshops: no data.  Suicide rates by sex: suicide rates (per 100,000) aged ≥ 65 years during implementation period in target area: 78 in women and 74 in men.  Change in suicide rates (aged ≥ 65 years) following the intervention, from baseline to implementation period: a statistically significant 76% reduction among women and a significant 73% reduction among men in target area compared with control. |
| 33 | Japan (Matsuno-yama) | Rural | 1977–1984 | 1985–1991 | Universal screening for everyone aged 65 years and over. | 59.8% aged 65–74 years, 32.3% aged 75–84 years and 7.9% aged 85 years and over during implementation period | No data. | Participation rate in screening, range of annual %: 92–97%, no data by sex.  Detection rate of depression, range of annual %: 1.5–3.9%, no data by sex.  Participation in workshops: no data.  Suicide rates by sex: suicide rates (per 100,000) aged ≥ 65 years during implementation period in target area: 168 in women and 30 in men.  Change in suicide rates (aged ≥ 65 years) following the intervention, from baseline to implementation period: a statistically significant 60% reduction among women and a significant 88% reduction among men in target area compared with control. |
| 34 | Japan (Yasuzuka) | Rural | 1981–1990 | 1991–2000 | Universal screening for everyone aged 65 years and over. | 60.2% aged 65–74 years, 31.7% aged 75–84 years and 8.1% aged 85 years and over during implementation period | Unemployment rate (0.44%) in Yasuzuka was much lower than prefectural mean (2.02%) in Niigata in 1990.  Average yearly income (2,340,000 yen) in Yasuzuka was 88.8% of prefectural mean (2,636,000 yen) in Niigata in 1990. | Participation rate in screening, range of annual %: 90–97%, no data by sex.  Detection rate of depression, range of annual %: 0.3–1.1%, no data by sex.  Participation in workshops: no data.  Suicide rates by sex: suicide rates (per 100,000) aged ≥ 65 years during implementation period in target area: 104 in women and 174 in men.  Change in suicide rates (aged ≥ 65 years) following the intervention, from baseline to implementation period: a statistically significant 64% reduction among women and a non-significant 49% reduction among men in target area compared with control. |
| 35 | Japan (Matsudai) | Rural | April 1978–March 1988 | April 1988–March 1998 | Universal screening for everyone aged 65 years and over. | 60.2% aged 65–74 years, 32.2% aged 75–84 years and 7.6% aged 85 years and over during implementation period | Unemployment rate (0.52%) in Matsudai was lower than prefectural mean (2.29%) in Niigata in 1985.  Average yearly income (1,879,000 yen) in Matsudai was 89.7% of prefectural mean (2,095,000 yen) in Niigata in 1985. | Participation rate in screening, range of annual %: 95–99%, no data by sex.  Detection rate of depression, range of annual %: 1.1–2.8%, no data by sex.  Participation in workshops: no data.  Suicide rates by sex: suicide rates (per 100,000) aged ≥ 65 years during implementation period in target area: 104 in women and 283 in men.  Change in suicide rates (aged ≥ 65 years) following the intervention, from baseline to implementation period: a statistically significant 70% reduction among women and a non-significant 2% increase among men in target area compared with control. |
| 36 | Japan (Nagawa) | Rural | 1993–1998 | 1999–2004 | Universal screening for everyone aged 65 years and over. | 55.6% aged 65–74 years, 35.6% aged 75–84 years and 8.7% aged 85 years and over during implementation period | No data. | Participation rate in screening, range of annual %: 80–95% in women and 60–89% in men.  Detection rate of depression, range of annual %: 0.5–2.3%, no data by sex.  Participation in workshops: no data.  Suicide rates by sex: suicide rates (per 100,000) aged ≥ 65 years during implementation period in target area: 125 in women and 193 in men.  Change in suicide rates (aged ≥ 65 years) following the intervention, from baseline to implementation period: a statistically significant 74% reduction among women and a non-significant 52% reduction among men in target area compared with control. |
